# Growing CAPITOL 4 Life: A Case Study of Co-designed Material for the First 1000 Days Using a Modified Double Diamond Approach

**DOI:** 10.1007/s10995-026-04320-w

**Published:** 2026-08-31

**Authors:** Andrew P. Hills, Sisitha Jayasinghe, Kylie Mulcahy, Kylie Burgess, Lisa Dalton, Kiran D. K. Ahuja, Roger Hughes, Nuala M. Byrne

**Affiliations:** 1 School of Health Sciences, UTAS Health, University of Tasmania, Launceston, 7250 Australia; 2 Independent Researcher, Newcastle upon Tyne, NE1 8SG United Kingdom; 3 Burnie Works, Burnie, TAS 7320 Australia; 4 Department of Nursing and Allied Health, Swinburne University of Technology, Hawthorn, Vic 3122 Australia

**Keywords:** Co-design, Double diamond, First 1000 days, Health promotion, Participatory design

## Abstract

**Introduction:**

Growing CAPITOL 4 Life is a place-based public health initiative focused on the first 1000 days (F1D), from conception to a child’s second birthday. It sought to strengthen community capacity for early-childhood health by leveraging local knowledge, assets and partnerships, while moving beyond one-way information provision towards sustained learning, empowerment and collective action. This study retrospectively examined the co-design process through the lens of the Double Diamond.

**Methods:**

Parents, educators, service providers and community stakeholders across Burnie, Circular Head and Devonport, Tasmania, participated in co-design workshops. Engagement spanned government, local government, Aboriginal organizations, libraries and community services. The process was facilitated by a place-based systems-change organization and supported by the University of Tasmania. Community members contributed lived experience alongside professional and academic knowledge, with decision-making remaining largely community-led and guided by the principle of “for the community, by the community.”

**Results:**

Across the four phases, communities moved from sharing experiences and identifying strengths and challenges (Discover), to refining priorities (Define), developing locally relevant resources and learning approaches (Develop), and shaping feasible delivery models (Deliver). The process centered on the F1D domains of Connection, Nutrition, Caring and Moving and resulted in community-informed Evidence Briefs, workshops and playgroup-based learning. Importantly, the Double Diamond was reflected not as a prescribed sequence, but as a recurring pattern of opening outward to explore and narrowing inward to make sense, prioritize and act. Discussions moved organically between what was working, what was missing, what an ideal future might look like and how communities could move towards it.

**Discussion:**

The findings suggest that the value of the Double Diamond in public health lies less in adherence to predefined stages than in providing sufficient structure for purposeful exploration while preserving community agency. This flexible approach may support locally relevant, acceptable and trusted solutions, particularly in complex regional settings where effective public health responses must emerge from, rather than be imposed upon, communities.

## Significance

What is Already Known on this Subject?

What this Study Adds?

F1D, spanning from conception to a child’s second birthday, represents a critical developmental window with lasting implications for health and well-being. Public health initiatives increasingly emphasize this period to prevent disease, promote healthy growth, and optimize potential.

This case study demonstrates that the Double Diamond co-design method can be effectively applied in small regional communities. It highlights the value of community-led decision-making, shared care principles, and context-specific implementation to enhance program acceptability, sustainability, and impact.

## Introduction

The first 1000 days (F1D) refers to the period from conception to a child’s second birthday (Darling et al., 2020). As a critical window for development with profound and potentially lasting impacts on health and well-being, public health initiatives recognize the importance of the F1D for preventing disease, promoting healthy development, and maximizing a child’s potential (Darling et al., 2020; Hills et al., 2025). Growing CAPITOL 4 Life is one such public health initiative, the success of which hinges on close collaboration between community members, researchers, and policymakers. Specifically, it is a place-based public health endeavor that built community capacity and collective action by leveraging local assets, knowledge and partnerships to improve health and support optimal early-childhood development. The overarching aim of this initiative is to strengthen community knowledge, confidence and capability to support children’s health and development throughout the F1D, moving beyond one-off information provision towards sustained learning, empowerment and community capacity. Briefly, across the three sites, this was conceptualized as a progressive pathway from knowledge, through discovery and application, to attainment, with parents and caregivers supported to translate evidence and lived experience into everyday practice, share learning within their networks, and increasingly become advocates and community champions. These outcomes were anchored in four interconnected foundations of early-life wellbeing -Connection, Nutrition, Caring and Moving - which provided a common framework for learning, discussion and action across the project.

Communities are often excluded or marginalized within dominant evidence chains, leading to their knowledge being perceived as less legitimate or rigorous. Amplifying community voices can strengthen partnerships and foster innovative solutions to complex challenges, paving the way for impactful and sustainable improvements in population health (Vargas et al., 2022; Verloigne et al., 2017). In this context, approaches using knowledge co-creation are increasingly valued as they bring together diverse stakeholders in a collaborative process of creative problem-solving (Clarke et al. 2017; Slattery et al. 2020a). This approach places a deliberate emphasis on directly engaging end users in developing and implementing intervention strategies tailored to specific settings (Greenhalgh et al., 2016; Voorberg et al., 2015). Notably, co-creation approaches have the potential to enrich the traditional evidence hierarchy by offering a more holistic and nuanced perspective, empowering end users to have a meaningful voice in their own health outcomes. This shift has been shown to enhance knowledge transfer in healthcare and substantially bridge the persistent gap between research and practice, representing a significant advancement toward more effective and equitable public health (Powell et al., 2018; Ward, 2017).

In terms like co-creation, co-design, co-production, and several other examples, the prefix “co-” before a verb is used to denote joint, collaborative, or shared action between two or more parties (Grindell et al., 2022). Although the intention is to signal that an activity is done collectively, often across different roles, sectors, or communities, the potential ambiguity created by these similar terms has been referenced. For example, Williams et al. used the term “cobiquities” to highlight the overabundance and sometimes diluted meaning of “co-” approaches (Williams et al., 2020). Co-creation, co-design, and co-production are related but distinct concepts, especially in participatory research, policy development and public health. *Co-creation* involves engaging stakeholders in the collaborative development of ideas, solutions, or knowledge between stakeholders (Leask et al., 2019; Ozcan & Ramaswamy, 2014). *Co-design* is focused on the design phase of a program, service, or intervention, where stakeholders collaboratively shape its features often drawing on co-created integrated knowledge translation. *Co-production* involves the collaborative implementation and delivery of services or knowledge production, where responsibilities and roles are shared (Vargas et al., 2022). To this end, and to reduce confusion, it is helpful to view co-design as a component within the broader themes of co-creation and co-production (De Koning et al., 2016; Jones, 2018; Vargas et al., 2022).

### Overview of Co-design

Co-design is an intentional process that requires a particular approach and facilitation skillset, seeks to involve stakeholders in a process to ensure that results meet the needs of the people for whom it is designed (Dawda & Knight, 2018). Specifically, co-design requires a partnership with end-users in the planning, design and evaluation of programs and promotes ownership and engagement by participants (Szebeko & Tan, 2010). This enables increased trust, participation and ownership by the community. Importantly, it reconceptualizes the role of health care consumers and ensures their involvement in all stages of improving the quality of health products and services (Robert et al., 2015).

Further, in co-design, experiential knowledge is valued and the process is underpinned by principles such as equality – all participants are equal, diversity – inclusion of a diverse range of stakeholders, and accessibility – all participants have equal opportunity to participate (Dawda & Knight, 2018). The benefits of using a co-design approach include services becoming more user-centred; releasing innovation; creating social capital; and empowering people to take more responsibility for their own well-being (Szebeko & Tan, 2010).

Co-design has been successfully used in a variety of clinical areas and in community development work (Benz et al., 2024; Calvo & Sclater, 2021; Donetto et al., 2014; Robert et al., 2015). To the best of our knowledge, we found no published examples comparable to the present approach, in which co-design was used to develop learning resources for a public health initiative focused specifically on the F1D. Accordingly, the main aim of this work was to develop a place-based and place-focused parent and caregiver program with stakeholders in North West (NW) Tasmania using a co-design approach. This paper outlines key aspects of the co-design process and provides details of the development of program material being implemented across communities in the region.

## Methods

### Ethics Approval

All procedures were approved by the Human Research Ethics Committee (Redacted) Network (30027) and were implemented in accordance with relevant guidelines and regulations stipulated in the Declaration of Helsinki. Both written and verbal consent was obtained from all participants.

### Context

The NW F1D project builds on the earlier CAPITOL (Community Action in Preventing and Improving Obesity and Lifestyle) initiative (https://www.utas.edu.au/health/community-programs/capitol), a community-led, place-based program developed to strengthen local capacity for obesity prevention on Tasmania’s North-West Coast. The strengths-based and place-focused approach established through CAPITOL provided the foundation for subsequent work focused on giving children the best start to life. Growing CAPITOL 4 Life represents this extension of the CAPITOL approach into the early-life period, with the NW F1D project forming a key component of this work. While the earlier CAPITOL initiative focused broadly on strengthening community capacity for obesity prevention, the NW F1D project applied these established community partnerships and principles specifically to the period from conception to two years of age. Thus, the present project was not a standalone intervention, but an evolution of an established place-based approach, adapted through community co-design to address local priorities for families and young children. The broader development of Growing CAPITOL 4 Life and its relationship to the earlier CAPITOL initiative have been described elsewhere (Hills et al., 2025).

During the CAPITOL project, the Burnie community identified as a major priority, the need to empower families to help all children reach their full potential. A joint venture between the collective impact organization, Burnie Works and the School of Health Sciences, University of Tasmania, secured funding to resource the substantive NW F1D project. Informed by the World Health Organization’s (WHO) Nurturing Care Framework for Child Well-being (Organization, 2021), the NW F1D project is centred on delivering key insights based on what the community identifies as vital knowledge for child-rearing and well-being. It is underpinned by the F1D quadrant model, which encompasses four interconnected domains: connection, nutrition, caring, and moving. These domains were originally created in Burnie through parental insights into early childhood nurturing. The NW F1D project expanded from Burnie to include Circular Head and Devonport local government areas (LGAs) and provide mothers and young children with the best start in life, promoting a healthy lifestyle through coordinated, evidence-informed community initiatives related to each quadrant. Blending local knowledge with evidence-based best practices has enabled the NW F1D project to generate outputs that include a range of co-designed resources, and a learning pathway developed in collaboration with parents, caregivers, and professionals.

The co-design process comprised of 12 facilitated workshops across three North-West Tasmanian communities - Burnie, Circular Head and Devonport- conducted between August and September 2024. Sessions were held in established community settings and brought together parents/caregivers and local service providers. Across sites, workshops progressed iteratively from establishing a shared vision and eliciting lived experiences, through examination and prioritization of the four F1D quadrants, to identification of community strengths, future aspirations, and actionable priorities. Evidence-informed materials, including quadrant-specific research videos, were used to stimulate discussion and connect evidence with lived experience, while participants collectively defined the project’s priorities, purpose and practical pathways for implementation. The co-design sessions incorporated structured elements of the Nominal Group Technique, particularly collective idea generation, discussion and prioritization, alongside iterative consensus-building processes characteristic of Delphi approaches.

### Co-design Logic Model

**Fig. 1:**
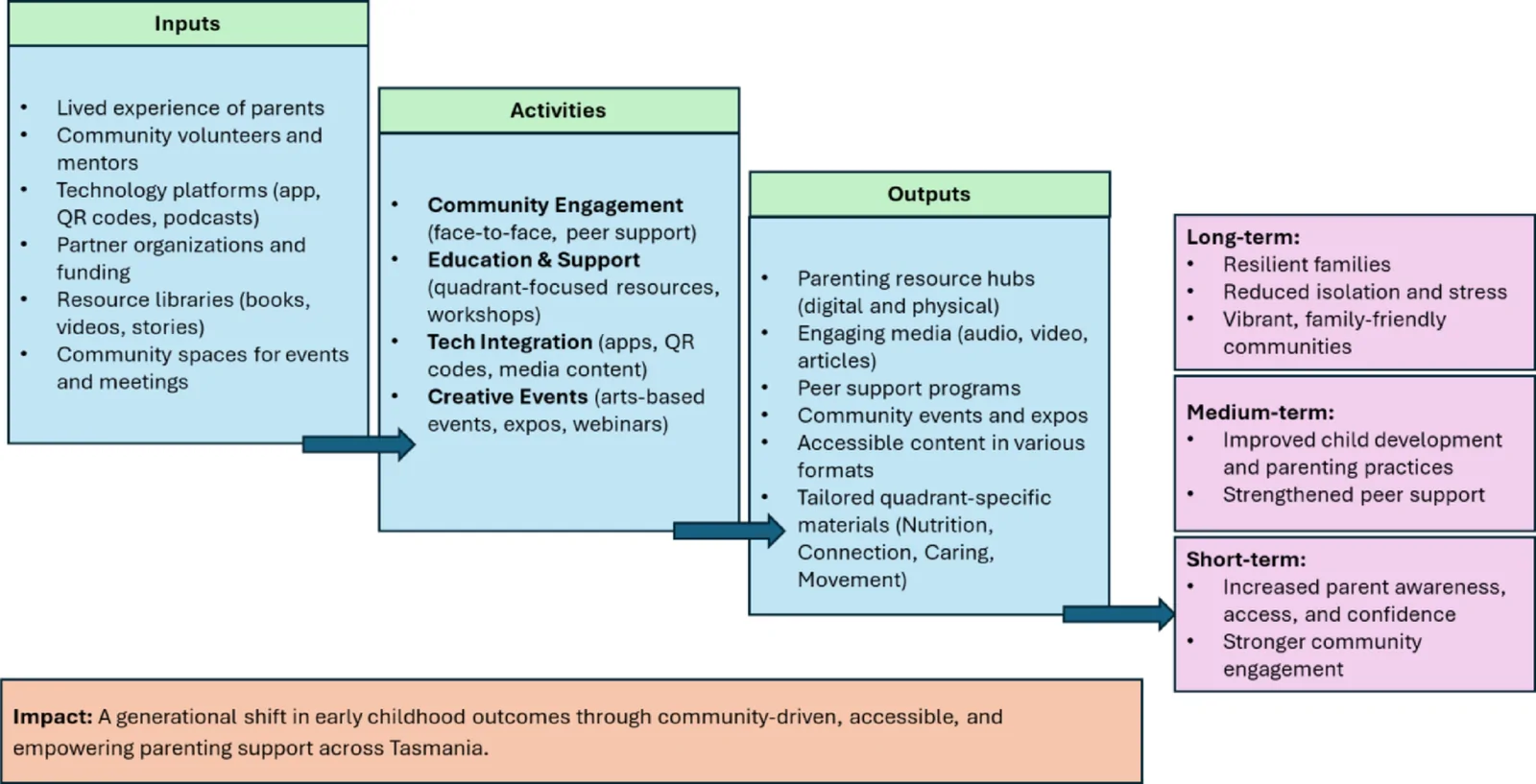
Logic model of the co-design initiative

The logic model provided an organizing framework for linking who and what was brought into the project (inputs), through the co-design and implementation activities, to the intended outputs and outcomes (Fig. 1). Rather than functioning as a prescriptive intervention pathway, it was used as a flexible framework to guide discussion, clarify the contribution of different stakeholders and resources, and ensure that activities developed through co-design could be connected to meaningful changes for families and communities. The inputs reflected the strengths already present within each community, including lived experience, community networks, partner organizations, existing resources and local infrastructure. These informed the co-design activities, through which communities shaped the educational, peer-support, digital and creative approaches represented in the model’s outputs. The subsequent outcomes provided a shared framework for considering how these locally developed activities could progress from increasing knowledge, confidence and engagement, to strengthening parenting practices, service connections and peer networks, and ultimately contributing to family wellbeing and early-childhood development. Importantly, the model therefore complemented rather than constrained the Double Diamond process: the Double Diamond provided a flexible process for exploring and developing solutions, while the logic model provided a framework for connecting those solutions to their intended contribution and impact. In this way, methodological decision-making remained community-led while retaining a clear line of sight between community assets, co-designed activities, implementation and longer-term public health goals.

### The ‘Double Diamond’ Approach

**Fig. 2:**
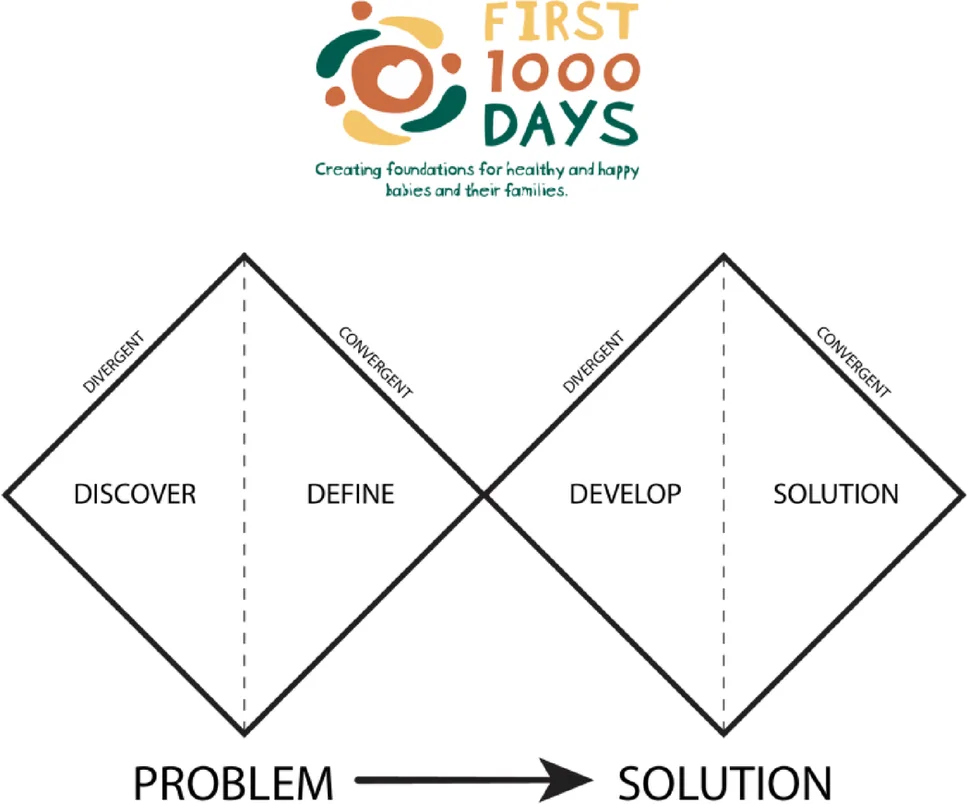
Growing CAPITOL 4 Life double diamond approach

The co-design elements of the project were based on the double diamond design-thinking process (Fig. 2) pioneered by the UK’s Design Council to help teams navigate complex problems and deliver innovative solutions (https://www.designcouncil.org.uk/our-resources/framework-for-innovation/). Although the advantages of this approach in implementing public health initiatives are well established (Brooks Carthon et al., 2021; Hawryszkiewycz & Alqahtani, 2020), its iterative structure- characterized by alternating phases of divergent thinking and convergent refinement- proved especially relevant in this context, facilitating the development of innovative and precisely targeted solutions.

## Results

### Co-design Process

A summary of site-specific co-design activities contextualized within the double diamond methodology, by region is presented in Table 1.

#### Phase 1–Discover

Established a shared foundation for open and respectful discussion across the three communities. Participants developed a collective Code of Care and subsequently shared personal stories and experiences of the F1D, including their interactions with organizations, health professionals and support services. These discussions revealed challenges at both individual and system levels, alongside locally identified opportunities and potential solutions. Participants actively questioned, refined and reframed these issues, resulting in a shared set of priorities and identified gaps in skills, knowledge and available support.

The co-design process was supported by a representative steering committee comprising researchers, service providers, parents and community representatives, including Aboriginal and Torres Strait Islander representation. The broader co-design team brought together project and research staff with participating parents, caregivers, community members and service providers, with community members contributing directly to priority setting, resource development and decisions about delivery. Across the sites, this structure supported a predominantly community-led process, reflecting the overarching principle of developing “a project for the community, by the community.” Indigenous perspectives were incorporated through the participation of Aboriginal and Torres Strait Islander representatives, parents, caregivers and service providers, with cultural safety, local knowledge and community preferences informing discussions and priorities.

#### Phase 2–Define

Participants collectively explored and refined the meaning, relevance and applicability of the four F1D quadrants-Connection, Nutrition, Caring and Moving- drawing on their own experiences and journeys. This process broadened the collective understanding of the support needs of families and highlighted the importance of creating safe, empathetic and supportive environments in which parents and caregivers could share experiences, discuss challenges and learn from one another. Rather than converging immediately on predetermined priorities, participants progressively identified the issues and forms of support they considered most meaningful for families and children.

#### Phase 3–Develop

This phase translated these priorities into tangible areas for learning and resource development. Participants identified baseline content across each of the four quadrants and endorsed the development of complementary Evidence Briefs to provide an accessible evidence base. Academic input helped identify appropriate evidence and existing resources, avoiding unnecessary duplication while ensuring that community-generated priorities remained central to resource development. Participants also identified peer learning and collective parenting wisdom as important mechanisms for engagement, consistently emphasizing the value of hearing how other parents navigated everyday challenges. Across sites, strengths-based discussions and the exchange of lived experiences emerged as particularly meaningful forms of learning. Participants further identified accessibility as a priority, favoring bite-sized resources, plain English and visual formats that could accommodate different levels of literacy and learning preferences.

#### Phase 4–Deliver

Two main implementation or delivery strategies were agreed upon by co-design participants:

1. *F1D Pregnant Parent Workshop Series*: grounded in the Code of Care to ensure a safe and supportive environment, these workshops feature guided discussions using story prompts and plain English resources that promote evidence-based best practices. Key insights and resource links will be visually recorded, and participants will receive take-home activities to reinforce learning. A structured feedback pathway will be available at each session for ongoing improvement.
2. *F1D Playgroup Series*: Led by playgroup facilitators, each session focuses on a specific F1D quadrant topic and includes guided exercises using visual prompts and simple language storytelling. Designed to encourage parent engagement, discussions invite open commentary while child activities are tailored to developmental needs. Facilitators will also provide best-practice resources and take-home activities when applicable.

**Table 1:** A summary of site-specific co-design activities contextualized within the double diamond methodology, by region

Region	Divergent & convergent phases		Divergent & convergent phases	
	Discover	Define	Develop	Deliver
Burnie	Gathered experiences of parents who understand the value of early parenting support through the F1 D framework and 4 quadrants: Connection, Nutrition, Caring, and Moving.	Clarified mission to spread accessible, relatable knowledge to parents, established a foundation of community-based learning and supports to boost parental confidence.	Engaged families and professionals to co-create innovative resources in diverse formats, from apps and podcasts to performances. Uses F1D Expo and quadrant-specific experts to support practical learning.	Agreed to deliver outcomes via various tools in multiple formats, ensuring accessibility and supporting quadrant needs like nutrition, emotional support, and safe movement, with a concerted focus on family support through lasting, community-based resources.
Circular head	Engaged parents, professionals, and community members to identify family needs and challenges in early childhood. Highlighted the importance of strong family support for a healthy, thriving community.	Established a mission to empower families with supportive networks, reliable information, and resources that reduce isolation and build confidence.	Invited parents, carers, and grandparents to co-create and test resources through events and online engagement, fostering family resilience and community growth.	Introduced refined solutions, supporting existing resources and filling gaps with new tools. Encouraged community participation to build a supportive environment for families in the F1D.
Devonport	Listened to parents and professionals, uncovered critical needs for guidance, community connection, and shared wisdom. Recognized that strong support leads to better family outcomes and futures.	Defined priorities such as building a network of parent mentors, creating a resource hub, and fostering empowerment through knowledge-sharing, focusing on a “village” approach.	Collaborated with the community to co-create resources aligned with the F1D timeline, addressing Nutrition, Connection, Caring, and Movement with diverse and inviting resources.	Agreed to deliver resources via user-friendly channels, including an app and in-person groups, emphasizing practical guidance on nutrition, connection, emotional regulation, and movement, with the overarching goal of strengthening community with holistic support.

### Co-design workshops

Twelve co-design workshops were conducted across the 3 sentinel sites (Table 2). The co-design sessions progressively moved from identification of strengths (i.e., what we do well?), all the way through to discussions about the ‘ideal future’ for each of the communities, and the ways in which these objectives could be realized (i.e., how?). Two illustrative examples from Devonport and Burnie are presented below.

**Fig. 3:**
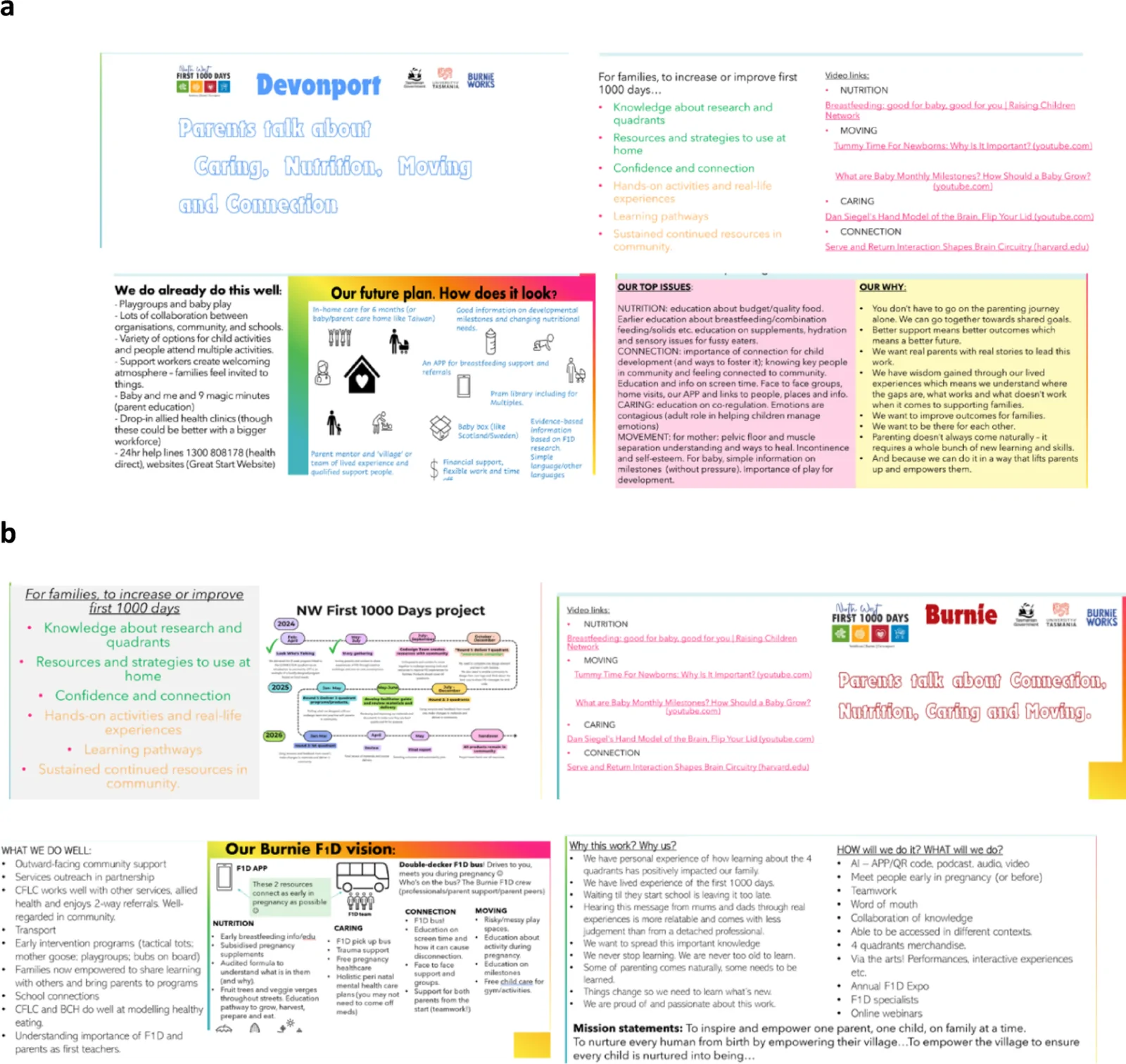
**a** Workshop story boards and discussions–Devonport. **b** Workshop story boards and discussions–Burnie

1. Devonport identified programs such as playgroups and baby play activities within the community to be reflective of a strong collaborative spirit among organizations and community members (Fig. 3a). Further, it was agreed that families benefit from a wide variety of child-centred activities that cater to diverse interests and developmental needs. The support workers’ role was also acknowledged as a strength in fostering an inviting atmosphere, making families feel welcomed and engaged. Programs like “Baby and Me” and “9 Magic Minutes” provide valuable parent education, supporting caregivers in building strong connections with their children. Additionally, drop-in allied health clinics offer accessible healthcare support, though the service would be enhanced with a larger workforce. Families also have access to 24-hour helplines, such as Health Direct, and resources like the Great Start Website, providing further guidance and assistance.
2. Burnie’s outward-facing community support and substantial outreach services were identified as significant strengths (Fig. 3b). Similarly, the well-established partnerships between the Child and Family Learning Centre (CFLC) and other services including allied health, and its 2-way referrals, were also identified as significant collaborative community endeavors. From a service provision perspective, the city’s transportation facilities were commended by all involved. Specifically, regarding early life programs, initiatives such as Tactical Tots, Mother Goose, Community playgroups such as Bubs on Board were highlighted as opportunities that enhanced family’s confidence and empowered them to share learnings with relevant others and expand the serviceability. From an organizational standpoint, the role of local schools and government entities such as the CFLC and Burnie Community House’s involvement in promoting and modelling healthy eating habits was also recognized. Further, the ongoing commitment from the community to appreciate the importance of the F1D and parents as first teachers was a main catalyst for community enthusiasm.

**Table 2:** Activity summary of co-design workshops, by region

Region	Date and time	Location	Attendance	Description
Burnie	5 August 2024,12:30 − 2:30 pm	CFLC	5 mothers, 1 father, 7 service providers	Project kick-off session introducing core objectives. Fostered a shared vision and participated in a logo consultation with Inclusive Creatives, establishing a cohesive project identity.
	12 August 2024, 12:30 − 2:30 pm (Session 1)	CFLC	4 parents, 5 service providers	Focused on elevating parent voices, with parents sharing insights and concerns. Research videos were presented for each quadrant, linking evidence-based insights to family experiences.
	12 August 2024 12:30 − 2:30 pm (Session 2)	CFLC	4 parents, 5 service providers	Afternoon session shifted to discussing current strengths and envisioning Burnie’s F1D future. Reflections on effective practices and outlining an ideal future state captured community hopes.
	16 September 2024, 12:30 − 2:30 pm	CFLC	4 parents, 7 service providers	Strategic session defining the project’s “WHY, HOW, and WHAT?” and crafting a mission statement. Finalized actionable next steps to align with the community’s vision for progress.
Circular head	13 t August 2024, 12 − 2 pm	Wyndarra Centre	4 mothers, 7 service providers	Project kick-off session introducing core deliverables and objectives. Parents shared voices and perspectives, contributing to a community-centered understanding. Attendees watched four videos related to quadrants, establishing an evidence-based foundation.
	3 September 2024, 12 − 2 pm	Wyndarra Centre	3 mothers, 5 service providers	Participants prioritized key issues for each quadrant through collaborative discussions and activities. They explored future aspirations, aligning the team’s efforts with clear priorities to advance community impact.
	10 September 2024, 12 − 2 pm	Wyndarra Centre	1 mother, 4 service providers	This session focused on strategic visioning, defining the project’s “WHY, HOW, and WHAT?” Participants established a purpose-driven roadmap that aligned with the project’s core goals, deepening commitment and clarity.
	24 September 2024, 12 − 2 pm	Wyndarra Centre	2 mothers, 3 service providers	Focus on identifying individual skillsets within the group and practical approaches for implementing the project’s “WHAT?” Discussion on creating a mission statement, reinforcing the team’s commitment to actionable goals and community empowerment.
Devonport	1 August 2024, 9:30 − 11:30 am	CFLC	2 mothers, 4 service providers	The initial session opened with a supportive space to elevate parents’ voices. Open discussions allowed parents to share insights, influencing project direction. A co-design session with Inclusive Creatives facilitated a collaborative logo design to resonate with all stakeholders.
	15 August 2024, 9:30 − 11:30 am	CFLC	3 mothers, 3 service providers	Participants discussed the project’s purpose and outcomes, aligning everyone on the vision. Research videos highlighted each quadrant, grounding the project in evidence and deepening understanding of each area.
	5 September 2024, 9:30 − 11:30 am	CFLC	3 mothers, 3 service providers	Focused on Devonport’s current strengths in the F1D initiative, participants reflected on successes and envisioned an ideal future aligned with community values and aspirations.
	11 September 2024, 9:30 − 11:30 am	CFLC	3 mothers, 1 father, 2 service providers	Final session explored top issues across quadrants, examining the “WHY, HOW, and WHAT?” of each concern. This discussion provided clarity for focused, meaningful actions in priority areas.

Importantly, the co-design process reflected the underlying logic of the Double Diamond, not as a prescribed sequence of steps, but as a flexible pattern of opening outward to explore, and then narrowing inward to identify and refine priorities. The Discover phase created space for communities to surface their experiences, strengths and challenges; Define progressively honed these diverse perspectives into shared priorities and understandings; Develop then opened the process again to generate, test and shape possible responses; and Deliver brought the process back towards a set of locally meaningful and feasible approaches. This pattern was evident across the workshops, where discussions moved organically between exploring what was already working, identifying gaps, imagining an ideal future and considering how that future might be realised. The value of the Double Diamond, therefore, lies less in following a fixed set of stages than in providing a scaffold for collective sense-making and iterative decision-making. Such an approach is particularly relevant to public health settings, where communities are complex, diverse and continually evolving, and where solutions cannot be meaningfully prescribed in advance. Rather, the process provides sufficient structure to support purposeful co-design while retaining the flexibility for communities to determine what matters, what is feasible and what solutions are most appropriate within their own context. Across four workshops at each site, the process moved from building relationships and shared understanding, through integrating lived experience with evidence, to collective visioning and priority setting, and ultimately towards locally owned strategies for action. This progression supported a shift from participation to leadership, with communities developing context-specific visions and increasingly taking ownership of initiatives, decision-making and delivery.

## Discussion

Importantly, the findings from this study suggest that co-design can offer a particularly valuable pathway for implementing public health initiatives within small, geographically and socially distinct communities. Rather than simply facilitating consultation, co-design has the potential to strengthen community ownership, generate contextually relevant solutions, build trust, and foster more sustainable approaches to public health practice (Campagnaro et al. 2021; Slattery et al. 2020b). The value of this approach may be particularly evident for initiatives involving children and families, where health, education, social support and community services are inherently interconnected. By creating accessible community-based spaces and “soft entry” points into support, co-design can help bridge formal services with the everyday experiences of families, while strengthening connections between practitioners, organizations and communities (Hall et al., 2023).

At the same time, our experience suggests that the success of co-design should not be viewed as an inevitable consequence of bringing stakeholders together. The process is relational and contingent, requiring skilled facilitation, adequate resources, safe and inclusive environments, and meaningful representation of the communities involved. While the NW F1D initiative benefited from these conditions, the broader literature highlights persistent structural barriers, including limited data sharing, restrictive funding arrangements, fragmented decision-making, inadequate local coordination and incentives that privilege tangible outputs over meaningful outcomes (Van Panhuis et al., 2014; Williams et al., 2020). These observations reinforce an important distinction: co-design is not simply a method for obtaining community input; it is a process through which communities are enabled to shape what matters, why it matters, and how challenges should be addressed. Its effectiveness therefore depends as much on the conditions created for participation as on the tools used to facilitate it.

The current project offers several significant insights that may inform future practice. Firstly, the diversity of stakeholders included in this co-design process is noteworthy. Key personnel involved represented not only the numerous unique contexts of each community but also the essential perspectives from administrative and health service sectors related to the F1D. By fostering a highly inclusive and representative approach, this method provided deeper insights into existing priorities, areas needing improvement, and effective solutions achieved by integrating end users and decision makers rather than isolating them, as is common in some public health research. Additionally, this inclusive strategy supports the formation of egalitarian partnerships that work to reduce disparities and promote fair treatment, ensuring all participants have equitable access to resources and respect for their contributions, regardless of their background. This approach further empowers stakeholders by fostering an environment where all voices are heard, active participation is promoted, and decision-making autonomy is preserved.

A further key advantage is the participatory nature of the approach which facilitates the creation, sharing, and integration of locally generated knowledge and lived experiences from diverse sources. This means that individuals whose life or work are the focus of the research actively engage throughout the research process, an essential principle of participatory research that was fully embraced in this program. Similar past studies have underscored the value of this democratic, equitable approach, highlighting how it leads to practical, contextually relevant, and locally adapted health promotion initiatives (Israel et al., 1998; Patton et al., 2003; Wright, 2013).

The iterative engagement among all stakeholders is also vital learning. One-off interactions among decision-makers and stakeholders are often inadequate, especially in matters of equality and social justice within public health (Hawkins et al., 2017; Southwick et al., 2021). Instead, this program involved a series of sessions, ensuring ample opportunity for stakeholders to collaboratively workshop the most effective strategies for their respective communities.

The site-specific trajectories highlight a central strength of the approach: each community was treated as a distinct social and contextual entity throughout the Double Diamond, rather than being expected to progress towards a predetermined solution. The divergent phases created space for communities to surface their own experiences, strengths and challenges, while the subsequent convergent phases enabled these insights to be collectively refined into locally meaningful priorities and actions. Consequently, the process unfolded differently across sites—Burnie building rapidly on established relationships and confidence, Devonport developing participation and leadership more gradually, and Smithton adapting engagement to local access, workforce and cultural contexts. This variation was not a limitation but an essential feature of the co-design process, demonstrating that effective co-design is not about imposing uniformity or seeking consensus at the outset, but about creating the relational and psychological space for communities to identify, interrogate and address their own priorities organically. In this sense, the Double Diamond provided more than a design framework; it enabled a continual process of “searching widely and honing in”, ensuring that solutions emerged from the community rather than being prescribed by the project team—arguably reflecting the fundamental meaning of co-design itself.

## Conclusions

This initiative provides a practical example of how carefully tailored co-design sessions can foster equitable participation, minimize the influence of dominant voices, surface diverse perspectives, and mitigate groupthink, thereby supporting efficient decision-making and prioritization. The use of creative techniques, including storyboards, sketches and drawings, further enriched the process by making lived experiences and ideas more tangible and accessible. Collectively, these approaches suggest that meaningful co-design can enhance the relevance, acceptability and trustworthiness of research within local communities. However, sustaining meaningful participation over the longer term remains challenging, with risks of tokenistic engagement alongside substantial demands on time and resources and uncertainty regarding translation into measurable health outcomes. These challenges may be mitigated through clear communication, well-defined roles and responsibilities, engagement of relevant decision-makers, and appropriate training for co-design facilitators. Importantly, while co-design is often confined to the planning phase of public health initiatives, this case study demonstrates how it can be embedded across the full project lifecycle, with the Double Diamond providing a practical framework for progressing from community-defined needs to the development and delivery of contextually relevant solutions.

## Data Availability

All relevant data are included in this manuscript. Additional information is available from the corresponding author upon reasonable request.
